# Anti-Biofouling Polymers with Special Surface Wettability for Biomedical Applications

**DOI:** 10.3389/fbioe.2021.807357

**Published:** 2021-12-07

**Authors:** Zhoukun He, Xiaochen Yang, Na Wang, Linpeng Mu, Jinyuan Pan, Xiaorong Lan, Hongmei Li, Fei Deng

**Affiliations:** ^1^ Institute for Advanced Study, Research Center of Composites and Surface and Interface Engineering, Chengdu University, Chengdu, China; ^2^ School of Mechanical Engineering, Chengdu University, Chengdu, China; ^3^ National Engineering Research Center for Biomaterials, Sichuan University, Chengdu, China; ^4^ School of Food and Biological Engineering, Chengdu University, Chengdu, China; ^5^ Department of Nephrology, Jinniu Hospital of Sichuan Provincial People’s Hospital and Chengdu Jinniu District People’s Hospital, Chengdu, China; ^6^ Department of Nephrology, Sichuan Academy of Medical Sciences and Sichuan Provincial People’s Hospital, University of Electronic Science and Technology of China, Chengdu, China

**Keywords:** anti-biofouling, antifouling, superhydrophilic, hydrophilic, hydrophobic

## Abstract

The use of anti-biofouling polymers has widespread potential for counteracting marine, medical, and industrial biofouling. The anti-biofouling action is usually related to the degree of surface wettability. This review is focusing on anti-biofouling polymers with special surface wettability, and it will provide a new perspective to promote the development of anti-biofouling polymers for biomedical applications. Firstly, current anti-biofouling strategies are discussed followed by a comprehensive review of anti-biofouling polymers with specific types of surface wettability, including superhydrophilicity, hydrophilicity, and hydrophobicity. We then summarize the applications of anti-biofouling polymers with specific surface wettability in typical biomedical fields both *in vivo* and *in vitro*, such as cardiology, ophthalmology, and nephrology. Finally, the challenges and directions of the development of anti-biofouling polymers with special surface wettability are discussed. It is helpful for future researchers to choose suitable anti-biofouling polymers with special surface wettability for specific biomedical applications.

## Introduction

**FIGURE 1:**
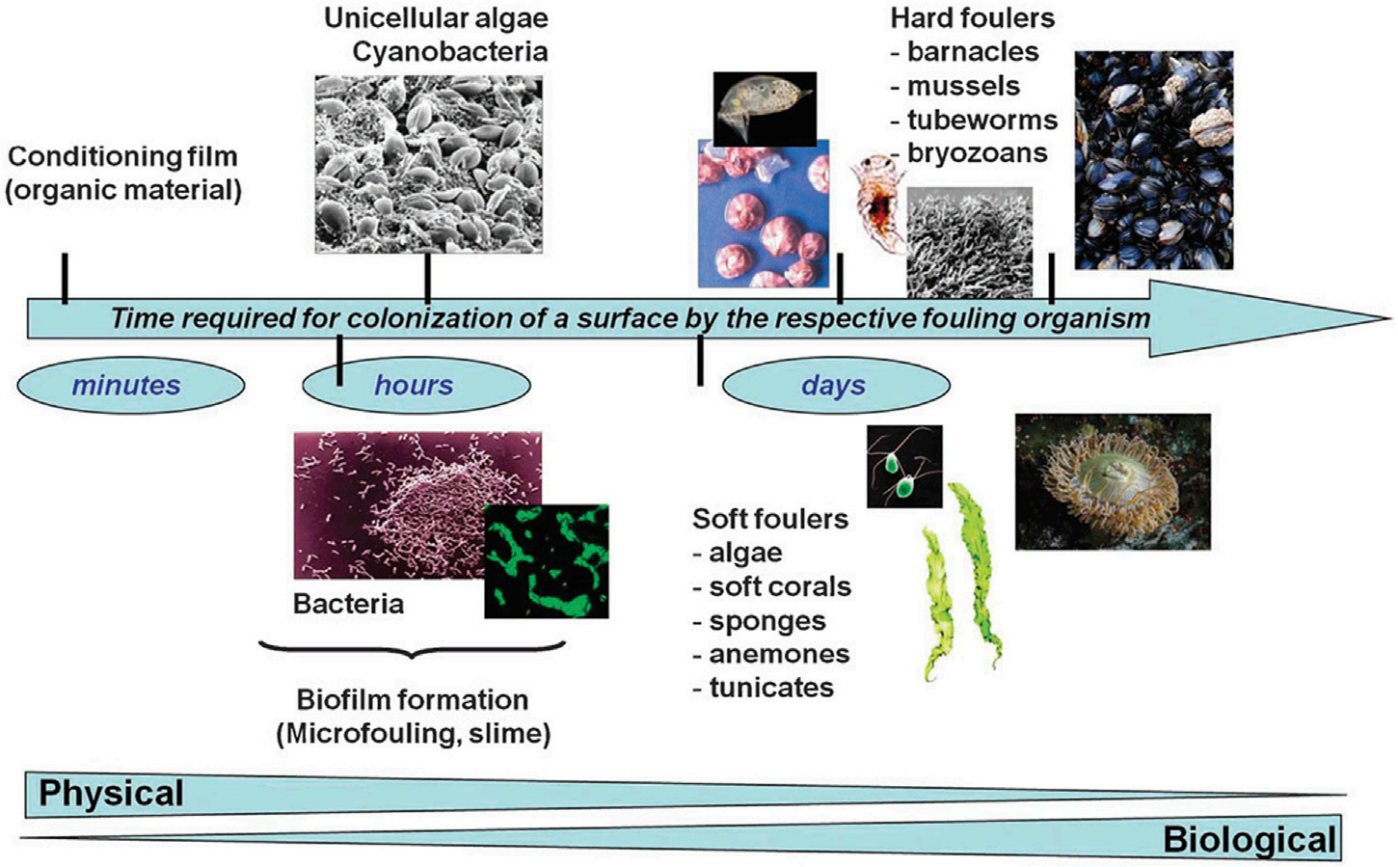
Surface colonization by a fouling organism. Reprinted with permission from Ref. (Rosenhahn et al., 2010). Copyright 2010, Royal Society of Chemistry.

The first known documentation of fouling is a papyrus dating from 412 BCE (1). To date, many kinds of fouling, such as dust, ice, crude oil, barnacles, bacteria, and blood, have been described and researched. Fouling has serious impacts on human life, as it degrades material surfaces, increases drag resistance in ships, and promotes infection in hospitals (1, (Almeida et al., 2007; He et al., 2021)). Our previous review defined four categories of foulant, namely, organic, inorganic, biofouling, and composite fouling (He et al., 2021). Biofouling is a persistent and widespread problem, the consequence of the aggregation of undesirable and often pathogenic organisms on surfaces, comprising biofilm produced by microorganisms and macroscale biofouling (macrofouling) resulting from foulants such as bacteria, cells, and proteins. As shown in Figure 1, biofouling usually begins with a surface film formed by organic molecules, to which different foulants attach, resulting in mixed communities that may undergo long-term changes over long periods of time (Rosenhahn et al., 2010). The presence of biofouling has significant impacts in various fields, including ships’ hulls, water pipes, biosensors, filters, and in the biomedical field where it contaminates applications such as surgical products, sutures, and dressings (Liu et al., 2020a).

Biofouling has been divided into three categories: marine, industrial, and medical (Callow and Callow, 2011; Bixler et al., 2014). In marine and freshwater environments, biofouling involves the undesirable attachment of organisms to artificial surfaces, such as ceramic, metal, or plastic (Dobretsov et al., 2013; Mieszkin et al., 2013; Hu et al., 2020). In the medical field, microorganisms may attach to devices and biosensors, resulting in the infection of patients (Jorge et al., 2012; Ammons and Copié, 2013; Leslie et al., 2014; Gaw et al., 2017). In industrial situations, microorganisms may feed and proliferate using nutrients in membranes, eventually blocking the pores (Bixler and Bhushan, 2012). Biofouling of microbes and viruses to surfaces, especially for medical biofouling, still remains an urgent problem to be solved owing to their crucial roles in medical implants, CLs, catheters, hemodialyzers, biosensors, and respirators (Jorge et al., 2012; Ammons and Copié, 2013; Leslie et al., 2014; Gaw et al., 2017). For example, the COVID-19 emergency lasted nearly 2 years but there is still no sign of it disappearing. The COVID-19 virus as a new kind of biofoulant is probably inhibited to fouling the materials with an anti-biofouling ability. Suhas S. Joshi and coauthors reported that fullerene-coated anti-biofouling surfaces could be a possible solution to decrease the adhesion of the COVID-19 virus on the surface, as they will be hydrophobic and toxic to the virus envelope (Siddiquie et al., 2020a).

**FIGURE 2:**
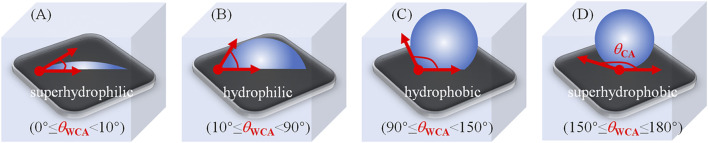
Diagrams of the degree of WCA ( $\theta_{WCA}$ ) and water droplets on the four surface types in air. Reprinted with permission from Ref from Ref. (He et al., 2021). Copyright 2021, Elsevier B.V.

The use of chemical coatings based on biocides or enzymes is the initial strategy in the prevention of fouling (Lejars et al., 2012). Although these strategies are effective in fouling prevention, they may be toxic to animals and plants in terrestrial and marine environments if there are some harmful materials in the coatings (Yebra et al., 2004), such as organotin, copper, etc. Because of this, there are restrictions and even prohibitions on the use of such materials. A following developed strategy is the use of self-polishing coatings. These rely on the hydrolysis of side-chains or degradation of the main polymer chain (Zhang and Chiao, 2015; Yang et al., 2020). Nevertheless, these coatings still have adverse environmental. Thus, the traditionally used chemical and self-polishing coatings are not adequate for the complex conditions present in the world today (Zhang et al., 2016). In biomedical situations, it has been proposed to use materials that either prevent the attachment of microorganisms to devices or destroy them in the vicinity of the device. These materials include coatings that repel or prevent attachment or kill the microorganisms in the vicinity. A variety of polymers have been developed to counteract or reduce biofilm (Carr et al., 2011; Jorge et al., 2012; He et al., 2021), including: *1*) cationic or peptide-mimicking polymers, or composites that can retain and release bioactive compounds; and *2*) systems that can prevent microbial attachment by either physical or chemical means. Antifouling and antimicrobial coating may be differentiated by ability of the former to repel microbes or modify the structure of biofilm, while the latter have either bacteriostatic or bactericidal activities. Antifouling coatings use steric repulsion or nanoscale rough topography to prevent microbial attachment, while antimicrobial materials interact directly, resulting in microbial death through physical contact or the release of bactericidal compounds (Zheng et al., 2021). In general, environmentally safe and non-toxic antifouling polymer coating materials thus require specific attributes of surface chemical compositions and physical structures, which both significantly affect the surface wettability that can be quantified as the water droplets contact angle (WCA, 0–180°) on the surface (Zhu et al., 2012; He et al., 2013; Tian et al., 2014; Yu et al., 2015; Kuang et al., 2016; Martin et al., 2017; Yu et al., 2018; Li et al., 2019a; Zhu et al., 2019). As defined by Young’s equation (Young, 1805), the Wenzel model (Wenzel, 1936), and the Cassie-Baxter model (Cassie and Baxter, 1944), the surface wettability can be described as superhydrophilic (WCA <10°), hydrophilic (WCA <90°), hydrophobic (WCA ≥90°), and superhydrophobic (WCA ≥150°), as shown in Figure 2.

**FIGURE 3:**
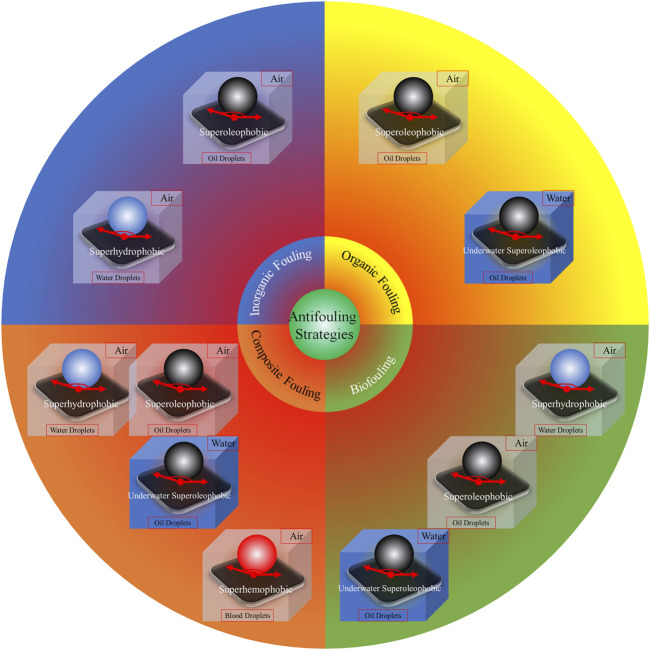
Antifouling strategies based on various super-phobic surfaces. Reprinted with permission from Ref. (He et al., 2021). Copyright 2021, Elsevier B.V.

Since the surface wettability and antifouling action of coatings are dependent on the properties, both chemical compositions and physical structures, of the surfaces (Maan et al., 2020), there should be some relationship between the surface wettability and antifouling ability of materials. Actually, many organisms with antifouling ability such as Lotus Leaf, Rice Leaf, and Shark Skin have a natural special surface wettability. After the design principle of materials with special surface wettability has been proposed by Lei Jiang et al. (Su et al., 2016), alterations in surface wettability allow the fine-tuning of bionic antifouling coatings and such techniques have attracted much attention over the past decade (He et al., 2021). The fluoro- and silicone-based hydrophobic polymers used in traditional antifouling materials reduce the attachment of the fouling substances to the surface (Lejars et al., 2012; Dobretsov and Thomason, 2011; Liang et al., 2020; Carl et al., 2012). Together with the chemical composition of the material, physical properties including the “Lotus Leaf,” “Rice Leaf,” and “Shark Skin” effects also influence the antifouling action (Zhang et al., 2016; Zhao and Liu, 2016; Shi et al., 2015; Lee and Yong, 2015; Roach et al., 2008; Pan et al., 2019; Jiang et al., 2015; Ball, 1999; Pu et al., 2016; Azemar et al., 2015; Kang et al., 2013; Bixler and Bhushan, 2013; Zhu et al., 2010; Bixler and Bhushan, 2014; Wu et al., 2011; Lee et al., 2013; Xia and Jiang, 2008). Engineered micro-topographical structures together with specific chemicals are commonly used for bionic implementation (Scardino and de Nys, 2011; Zarghami et al., 2019). Jie Zheng and coauthors have reviewed hydrophilic non-fouling materials and emphasized the importance of using strongly hydrated groups with optimal physical attributes on the material surface, concluding that, together with methods for coating surfaces, are critical for the development of stable and successful non-fouling materials for use in biomedical devices and applications (Chen et al., 2010). As shown in Figure 3, we have comprehensively reviewed the antifouling strategies for the four types of fouling according to different super-phobic surfaces, namely, superhydrophobicity in air (He et al., 2011; Martin and Bhushan, 2017), superoleophobicity in air (Chen et al., 2019; Li et al., 2020), superhemophobicity in air (Movafaghi et al., 2017; Galante et al., 2020), and underwater superoleophobicity (Du et al., 2017; Su et al., 2018).

In this review, we will focus on the anti-biofouling strategies, because the removal of fouling resulting from the deposition of organic or inorganic material is usually easier than eliminating biofouling. Superhydrophobic or superoleophobic surfaces are often able to prevent and release inorganic fouling, while superoleophobic surfaces or surfaces with underwater superoleophobicity are suitable for organic contaminants. Biofouling, however, usually involves a variety of foulants, and the solution is not simple. The most effective method, in terms of both cost and efficacy, is the use of surface wettability to counteract the attachment of foulants. Actually, an anti-biofouling surface can be achieved by tuning its surface wettability (Krishnan et al., 2008; He et al., 2021). For instance, anti-biofouling measures directed against bacteria rely on hydrophilic or superhydrophobic effects (Liu et al., 2020b). In addition, surfaces with superhydrophobicity, usually containing a layer of air that blocks contact between the foulant and the surface (Simovich et al., 2020), are effective against fouling, as shown in Figure 3.

Biofouling extensively exists in biomedical applications both *in vivo* and *in vitro*, such as cardiology, ophthalmology, nephrology, and various surgical equipment. Bacteria, cells, and proteins usually adhere to and forming dense collagenous capsule around the biomedical implants, which would induce inflammatory responses, and may give rise to infection and/or implant rejection (Chan et al., 2020). Thus, in view of the necessity for anti-biofouling in biomedical applications, we will focus on the anti-biofouling strategies based on polymers with special surface wettability such as superhydrophilicity, hydrophilicity and hydrophobicity but excluding superhydrophobicity which can be found in our previous review (He et al., 2021). Meanwhile, we just focus on the biomedical applications in cardiology, ophthalmology, and nephrology. Moreover, biofoulants mentioned in this review are focused on the usual bacteria, cells, and proteins. Although there are some reviews about antifouling polymers (Lejars et al., 2012; Wu et al., 2019; Maan et al., 2020), our review is focusing on anti-biofouling polymers with special surface wettability, and it will provide a new perspective to promote the development of anti-biofouling polymers. Meanwhile, the anti-biofouling strategies reviewed in this manuscript will offer help for future researchers to choose suitable polymers for specific anti-biofouling applications.

## Anti-biofouling Polymers With Special Surface Wettability

Anti-biofouling polymers are attractive as they can avoid the introduction of drugs to achieve anti-biofouling but their efficacy is determined by the polymer and foulant species (Francolini et al., 2015). As summarized in Table 1, numerous proposals with various surface wettability have been reported to generate anti-biofouling ability. The various types of polymers and biofoulants are also listed in Table 1, which clearly reveals the relationship between the polymers and foulants (bacteria, cells, proteins, etc.).

**TABLE 1:** A summary of polymers with different surface wettability and the relevant types of foulants for anti-biofouling applications (sorted in alphabetical order of the description of the typical polymers for each special surface wettability).

Strategy based on surface wettability	WCA(°)	Typical polymers	Foulants	Ref
Superhydro-philicity	0	MI-dPG	(a) Bacteria: *E. coli*, *S. aureus*	Li et al. (2019b)
	<5	PCBAA	(a) Cells: GLC-82 cells	Xu et al. (2017)
			(b) Proteins: FITC-BSA, FITC-HSA	
			(c) Blood: Blood cells, Blood proteins	
	0	Pluronic^®^ F127	(a) Cells: L929	Zheng et al. (2010)
			(b) Proteins: Fibrinogen, BSA.	
	0	PPGL	(a) Proteins: Anti-BSA, Anti-myoglobin	Gam-Derouich et al. (2011)
	6	Sulfobetaine silane	(a) Bacteria: *P. aeruginosa*, *S. epidermidis*	Yeh et al. (2014)
			(b) Proteins: BSA, Mucin, Lysozyme, Liposomes	
	10	Zwitterionic bottlebrush polymers	(a) Bacteria: *E. coli*	Xia et al. (2019)
			(b) Proteins: BSA, Lysozyme, β-Lactoglobulin	
	7	Zwitterionic hydrogels	(a) Bacteria: *S. aureus*, *E. coli*	Chan et al. (2020)
			(b) Cells: Human primary dermal fibroblasts, Red blood cells	
			(c) Proteins: BSA	
			(d) Blood: Platelets	
Hydrophilicity	28	PAA	(a) Proteins: BSA	Lei et al. (2021)
			(b) Cells: L929 cells	
			(c) Blood: Blood erythrocytes	
	27	PCBAA	(a) Bacteria: *E. coli*, *S. aureus*	Wang et al. (2018); Zhang et al. (2021a)
			(b) Cells: L929	
			(c) Protein: BSA, HRP-conjugated anti-IgG	
	26–74	PEG	(a) Bacteria: *S. epidermidis*, *S. aureus*, *P. aeruginosa*	Cheng et al. (2015); Wang and He, (2019)
			(b) Cells: Human corneal epithelial cells	
			(c) Proteins: BSA, Lysozyme	
			(d) Fungi: C. albicans, F. solani	
	41	PEGDA	(a) Blood: Platelet-rich-plasma	Guo et al. (2019)
	41	PEGylated	(a) Cells: NIH 3T3 cells	Chen et al. (2015a)
			(b) Protein: BSA.	
	58	PEO	(a) Bacteria: *S. aureus*	Martínez-Gómez et al. (2015)
	60	PHEMA	(a) Protein: BSA	Zhu et al. (2015)
			(b) Blood: Platelet.	
	25	Pluronic^®^ F127	(a) Cells: L929	Zheng et al. (2010)
			(b) Proteins: Fibrinogen, BSA.	
	72	Poloxamers 338	(a) Bacteria: *E. coli*	Stirpe et al. (2020)
	17	Poly(carboxylbetaine-co-dopamine methacrylamide) copolymer	(a) Bacteria: *E. coli*, *P. aeruginosa*, *S. aureus*	Liu et al. (2020c)
			(b) Fungi: C. albicans	
	56	Poly (citric acid)	(a) Proteins: BSA	Abidin et al. (2016)
	36	Poly(p-phenylene terephthalamide)	(a) Bacteria: *E. coli*	Chen et al. (2018)
			(b) Proteins: BSA	
	12–38	PSBMA	(a) Bacteria: *E. coli*, *S. epidermidis*	Chen et al. (2012); Sin et al. (2014); Li et al. (2017); Zhang et al. (2021b)
			(b) Cells: Human MG63 osteoblast, HT1080 fibroblast, L929	
			(c) Blood: Plasma protein, Blood platelets, Blood erythrocytes, Blood leukocytes	
	63	PVA	(a) Proteins: BSA	Lan et al. (2021)
			(b) Cells: L929 cells	
	20–60	PVP	(a) Bacteria: *S. aureus*, *E. coli*	Telford et al. (2010); Ran et al. (2011); Wu et al. (2012); Jiang et al. (2013); Liu et al. (2013); Zhu et al. (2017)
			(b) Cells: L929 cells	
			(c) Proteins: FITC-BSA, Fibrinogen, IgG, Lysozyme	
			(d) Blood: Platelets	
	85	Segmented PU with -SO_3_H	(a) Bacteria: *S. epidermidis*	Francolini et al. (2012)
	25–80	Others	(a) Bacteria: *S. aureus*, *E. coli*	Huang et al. (2011); Seo et al. (2011); Chen et al. (2014); Li et al. (2014); Xie et al. (2015); Yin et al. (2015); Wang et al. (2016); Valencia et al. (2018); Xie et al. (2018); Ji et al. (2019); Ye et al. (2019)
			(b) Cells: L929 cells, Bovine aortic endothelial cells	
			(c) Proteins: BSA, Fibrinogen, Lysozyme	
			(d) Blood: Platelet-rich plasma, Platelet-poor plasma	
Hydrophobicity	125	2-perfluorooctylethyl methacrylate	(a) Proteins: FITC-BSA, Fibrinogen	Wang et al. (2015)
	100–147	PDMS, PU, silicone oil	(a) Bacteria: *E. coli*	Siddiquie et al. (2020b)
	101	Poly(siloxane-urethane)	(a) Proteins: BSA.	Santiago et al. (2016)
	106	Others	(a) Bacteria: *S. aureus*, *E. coli*	Kim et al. (2016); Wang et al. (2017)
			(b) Proteins: BSA, Lysozyme	

### Superhydrophilicity

Surfaces that prevent both microbial attachment and non-specific protein adsorption are required in the biomedical sphere. These should be hydrophilic as the polymer surface should bind water in preference to microorganisms. Wetting is thus an important consideration (Chen et al., 2010). According to these criteria, a number of anti-biofouling polymers have been developed that effectively prevent the adhesion of proteins, cells, and bacteria. These include hydrophilic polymers (Epstein et al., 2012; Keefe et al., 2012; Chen et al., 2015a; Mohan et al., 2015; Zhu et al., 2015; Guo et al., 2019; Jiang et al., 2020), e.g., PEG, PEGylated polymers, PHEMA, polysaccharides, and zwitterionic polymers (Carr et al., 2011; Chen et al., 2012; Sin et al., 2014; He et al., 2016; Kang et al., 2016; Wang et al., 2018; He et al., 2019; Liu et al., 2020c; Erathodiyil et al., 2020; Su et al., 2020; Zhang et al., 2020; Zhang et al., 2021a; Zhang et al., 2021b; Zhou et al., 2021), e.g., PSBMA, PCBMA, and PCBAA. Although these polymers differ in their structures and chemistry, they are all able to bind strongly to water, resulting in the presence of a layer of water that reduces interaction and attachment between the surface and the foulant. Effective surface hydration is achieved through hydrogen bonding in the case of hydrophilic polymers, and ionic solvation in the case of zwitterionic materials (Liu et al., 2020a). In addition, some papers have been published on the synthesis and application of polyglycidol and its derivatives with various morphologies. For example, the PPGL with a WCA near zero showed a superhydrophilic character and good anti-biofouling ability tested in anti-BSA and anti-myoglobin experiments (Gam-Derouich et al., 2011).

**FIGURE 4:**
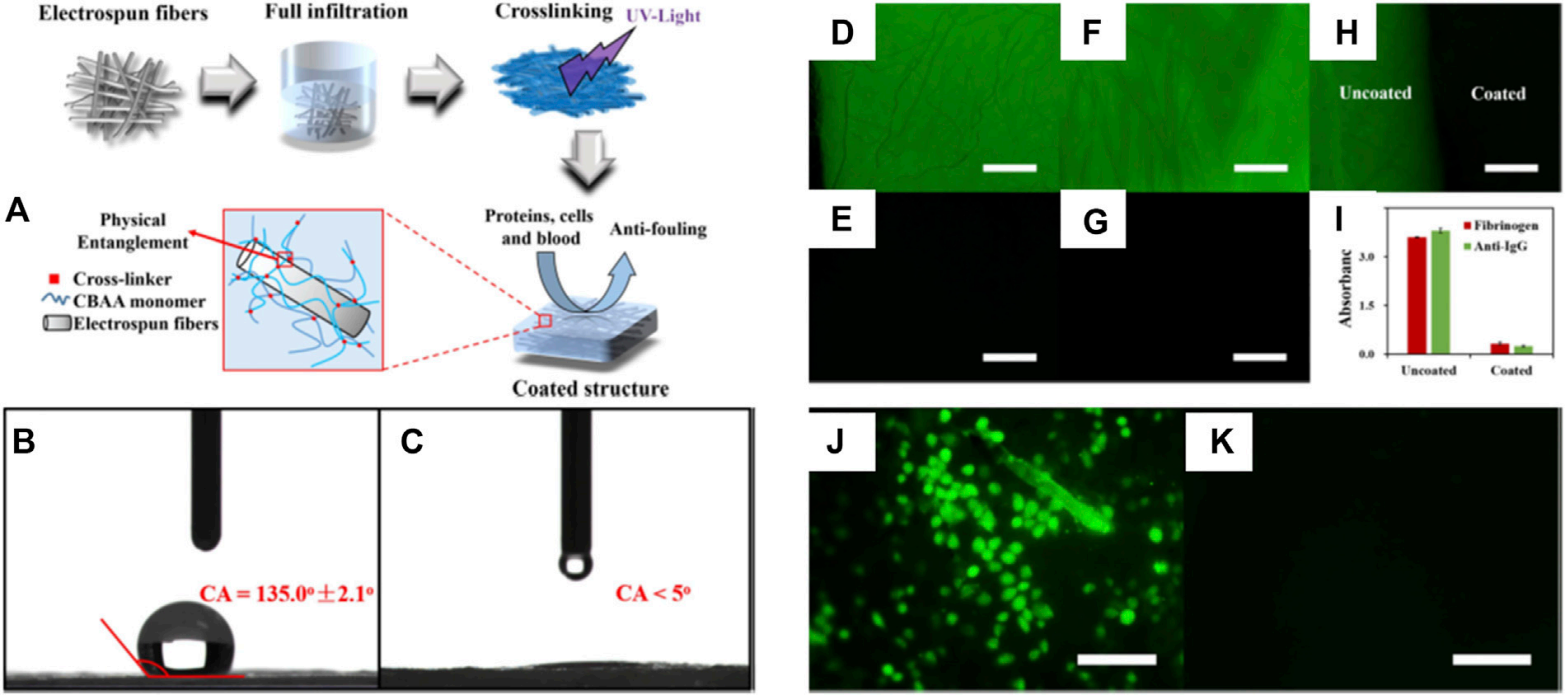
The process of preparing anti-biofouling zwitterionic hydrogels **(A)**. WCA on uncoated mesh **(B)** and coated superhydrophilic mesh **(C)**. Fluorescence micrographs of FITC-BSA **(D**,**E)** and FITC-HSA **(F**,**G)** adsorption on uncoated **(D**,**F)** and coated structures **(E**,**G)**, respectively. Merge of uncoated and coated materials after FITC-BSA adsorption **(H)**, and the enzyme-linked immunosorbent assay (ELISA) results **(I)**. Fluorescence micrographs of cell adhesion test on uncoated mesh **(J)** and coated superhydrophilic mesh **(K)**. Scale bar = 100 μm. Reprinted with permission from Ref. (Xu et al., 2017). Copyright 2018, IOP Publishing, Ltd.

Superhydrophilic anti-biofouling zwitterionic polymers show great potential for biomedical applications (Chan et al., 2020; Xu et al., 2017; Xia et al., 2019). Rongxin Su and coauthors reported an anti-biofouling three-block polymer with zwitterionic chains on the bottlebrush polymers that showed high stability in high-saline solutions and over an extensive pH range (Xia et al., 2019). The anti-biofouling properties benefited from a low WCA near 10° as demonstrated by serum albumin and lysozyme adsorption with ultralow fouling properties of lower than 0.2 ng cm^−2^ (Xia et al., 2019). Jackie Y. Ying and coauthors produced a novel superhydrophilic anti-biofouling, biocompatible hydrogel formed by the crosslinking of polymers with calcium and monomers of methacryloyl-L-lysine (MLL), a zwitterionic amino acid (Chan et al., 2020). The resultant hydrogel containing 30% MLL was found to be strongly porous with a high degree of water encapsulation. The WCA on a glass slide with hydrogel coating decreased to 7.6° and the superhydrophilic hydrogel was effective in preventing bacterial, cell, and protein adhesion. The anti-biofouling hydrogel did not form capsules when subcutaneously implanted in mice over 2 months (Chan et al., 2020). Lei Zhang and coauthors reported an efficient and simple strategy (Figure 4A) to modify hydrophobic electrospun meshes with zwitterionic PCBAA hydrogels to obtain superhydrophilic anti-biofouling meshes with WCAs of less than 5° (Figures 4B,C) (Xu et al., 2017). The coated superhydrophilic mesh resisted attachment of FITC-BSA, FITC-HSA proteins, and GLC-82 cells (Figures 4D–4K). Furthermore, the hydrogel structure retained its stability under physiological conditions for a minimum of 3 months. This report demonstrates an effective technique for modulating hydrophilic surfaces on different fibrous structures, and may have widespread biomedical applications.

### Hydrophilicity

Among hydrophilic polymers, PEG-based polymers are probably the most investigated for biomedical applications, as PEG is both non-immunogenic and anti-thrombogenic, as well as being largely resistant to protein adsorption. The anti-biofouling action of PEG-based polymers is the result of both steric and hydration effects and is dependent on the size, branching, and surface-packing density of the specific PEG molecule (Francolini et al., 2015). A high degree of hydration on the surfaces of the polymers is necessary for effective anti-biofouling actions, although the molecular mechanisms and details involved are not fully understood. Jie Zheng and coauthors conducted a computational investigation of the properties of four poly(N-hydroxyalkyl acrylamide) (PAMs) brushes with different carbon spacer lengths (CSLs = 1, 2, 3, and 5) using molecular mechanics (MM), Monte Carlo (MC), and molecular dynamics (MD) simulations (Liu et al., 2020a). MM assessed the type of packing structure of the brushes, while MC simulations were used to evaluate the interaction between the brushes and a lysozyme, and MD was utilized for examining the interactions between the brushes, proteins, and water molecules. The results showed that minor variations in the CSL structure are able to influence both the surface hydration and antifouling properties of the surface, confirming experimental findings using surface plasmon resonance and sum frequency generation vibrational spectroscopy, as well as measurements of contact angles. These results promote improved understanding of PAM brushes and their properties in relation to anti-biofouling materials and surfaces (Liu et al., 2020a).

Polyurethane (PU) is a commonly used biocompatible polymer, being used in numerous biomedical engineering applications, including dressings, joints, and catheters. Adsorption of proteins to its surface frequently occurs in biological and medical situations, with consequent deleterious effects. Thus, investigation of PU-protein interactions is critical. Both the chemical constituents of the material and its physical topography influence adsorption, and these factors have been extensively investigated over the past few decades. These studies have demonstrated the efficacy of tethering hydrophilic polymers, such as PEO, to the surface. Surfaces modified in this way are strongly resistant to nonspecific protein adsorption, with both the lengths and densities of the PEO chains playing significant roles (Zheng et al., 2010).

**FIGURE 5:**
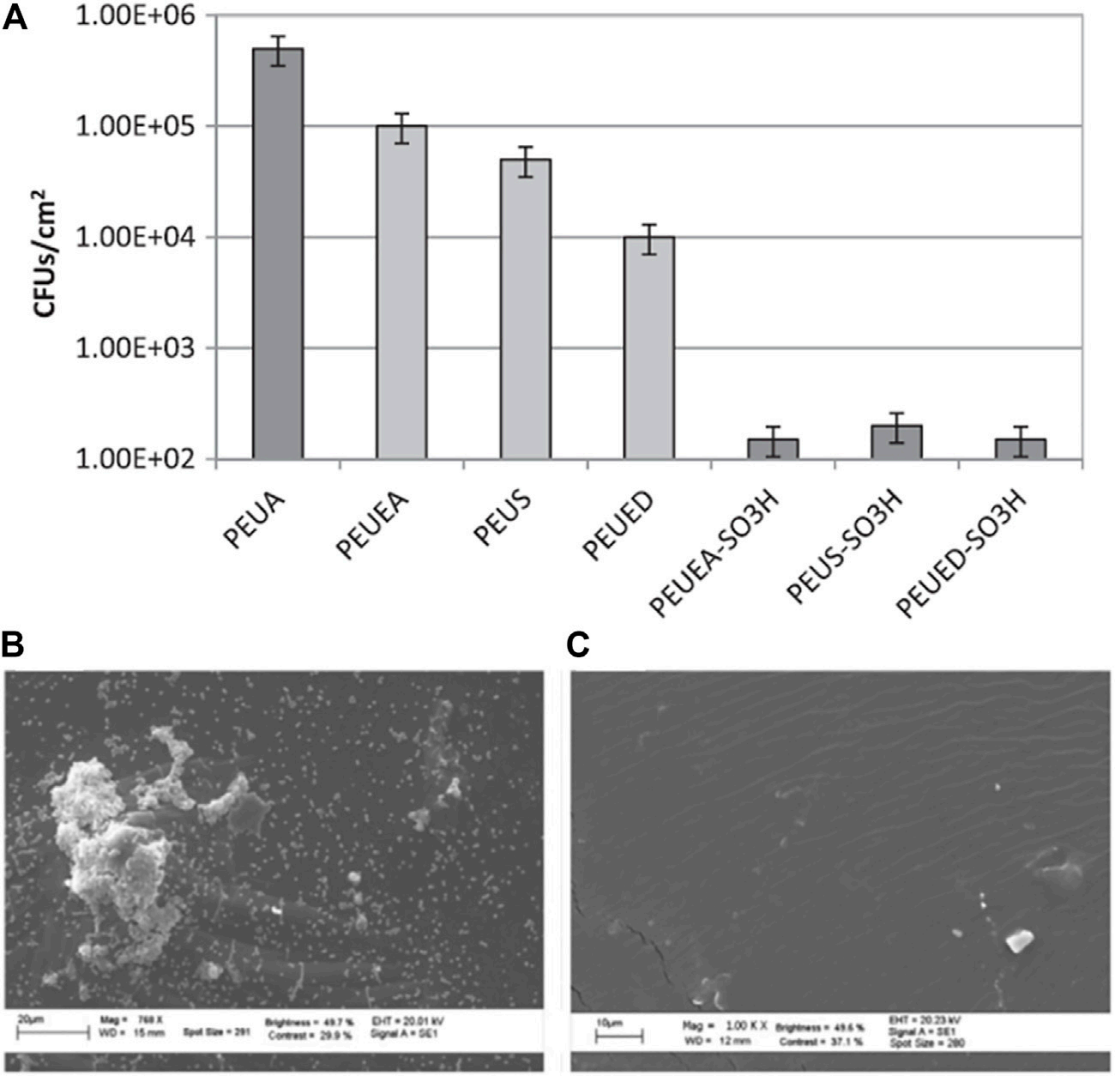
*S. epidermidis* colony-forming units per cm^2^ of PEUA (control), amidated, and -SO_3_H group-containing polymers **(A)**. SEM results showing the aggregates of bacterial on the PEUA surface **(B)** and the absence of aggregates on the PEUEA–SO_3_H surface **(C)** after 24 h of exposure. Reprinted with permission from Ref. (Francolini et al., 2012). Copyright 2012, Elsevier B.V.

In their investigation of suitable materials for preventing the complications of infection and thrombosis in devices making contact with the blood, Francolini and coauthors designed and synthesized a heparin-mimetic segmented PU (Francolini et al., 2012). This introduced sulfate and sulfamate moieties that are responsible for the anticoagulant activity of heparin onto PU. It was found that the modified PU was more hydrophilic than the parent compound. These polymers also reduced the degree of bacterial attachment, measured as colony-forming units (CFUs) found per cm^2^ of polymer (Figure 5A). These observations were confirmed by SEM (Figures 5B,C), which demonstrated bacterial colonization and aggregation on surfaces lacking the -SO_3_H groups (Figure 5B), and no accumulation of bacteria on surfaces with the -SO_3_H groups (Figure 5C). These results show that increasing the hydrophilicity of the polymer as well as the addition of -SO_3_H groups affected the antifouling action of the surface (Francolini et al., 2012).

### Hydrophobicity

Hydrophilic anti-biofouling polymers tend to swell, resulting in lower space for interaction and thus reduced attachments (Rosenhahn et al., 2010; Eshet et al., 2011). The use of hydrophobic polymers can evade this issue. Hybrid poly(siloxane-urethane) copolymers were developed by Lourdes Irusta and coauthors using isophorone diisocyanate trimers, polycaprolactone triols, and hydroxy-terminated PDMS (Santiago et al., 2016). The authors then used quartz crystal microbalance with dissipation monitoring to measure BSA adsorption, observing that the protein was adsorbed in a conformation that did not allow water retention. This indicates that the increased surface hydrophobicity produced by the PDMS was responsible for the improved antifouling action of these copolymers (Santiago et al., 2016). Xinping Wang et al. described the preparation of acrylate block polymer brushes with two 2-perfluorooctylethyl methacrylate units at the brush end on an Au substrate with a “grafting to” method (Wang et al., 2015). It was found that the amount of fibrinogen adsorbed to the surface was reduced in proportion to the hydrophobicity of the perfluoroalkyl chains (Wang et al., 2015).

**FIGURE 6:**
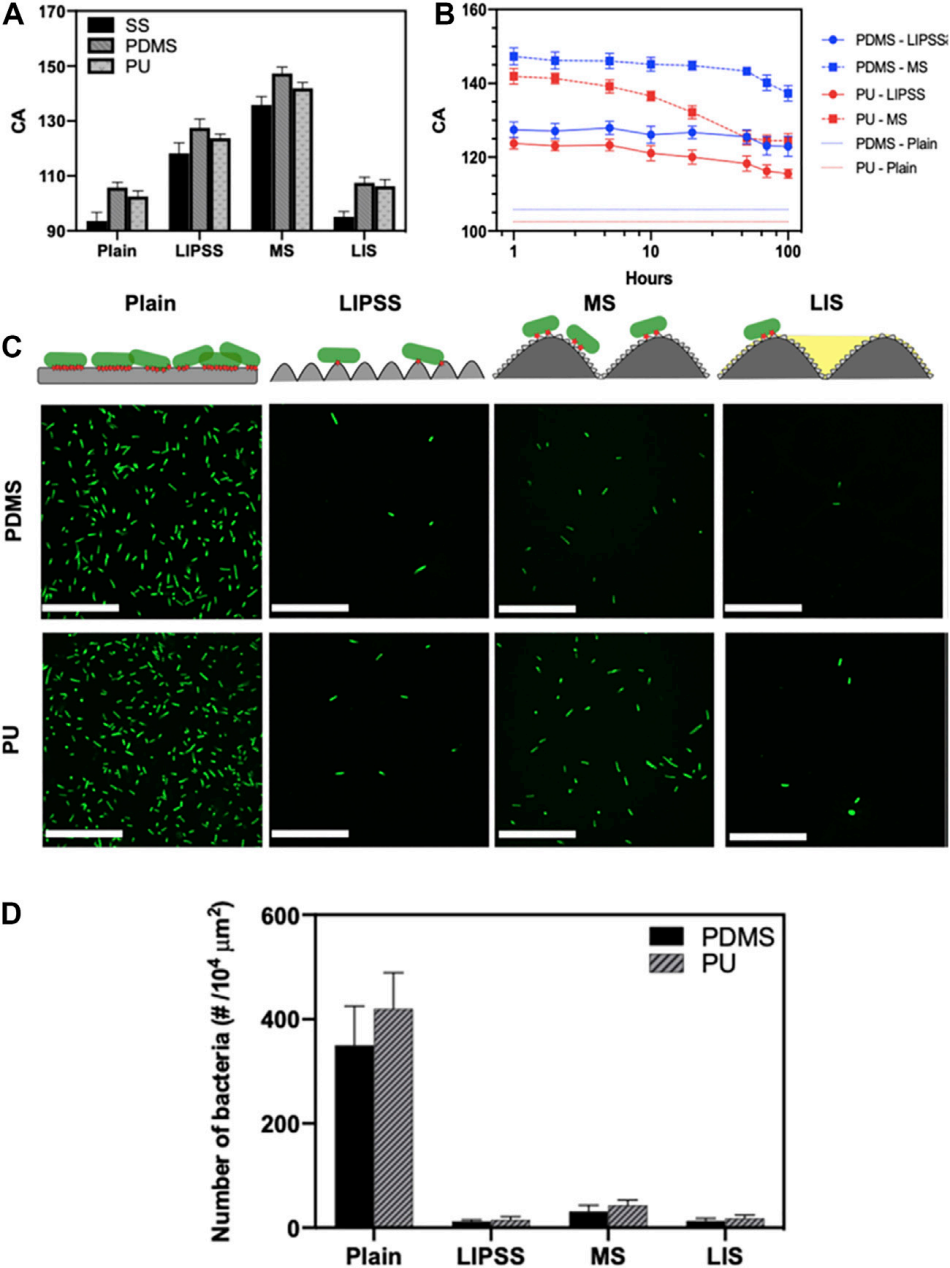
Static WCA on unmodified, LIPSS-, and MSs-modified SS, PDMS, and PU surfaces **(A)**. Differences in water CA with different immersion times **(B)**. Bacteria on the different surfaces (**C**, above). Bacterial contacts on the surfaces are shown in red, and fluorescence micrographs indicate bacterial attachment to different PDMS and PU surfaces (below). Numbers of attached bacteria in relation to topography **(D)**. Reprinted with permission from Ref. (Siddiquie et al., 2020b). Copyright 2020, American Chemical Society.

Suhas S. Joshi and coauthors investigated the effects of introducing femtosecond laser-induced submicron physical structures onto PDMS and PU surfaces for biomedical applications, as shown in Figure 6 (Siddiquie et al., 2020b). Highly regular and single scale submicron laser-induced periodic surface structures (LIPSS), and multiscale structures (MS) containing both submicron- and micron-scale features were obtained by femtosecond laser processing on stainless-steel (SS) substrates and following replicate processing with PDMS and PU elastomers. Surface hydrophobicity was enhanced on LIPSS and MSs surfaces (Figure 6A). It was observed that the hydrophobic submicron-textured PDMS and PU surfaces were stable and performed well for up to 100 h when immersed (Figure 6B). *E. coli* attachment was significantly reduced (>89%) on both LIPSS- and MS-modified surfaces (Figures 6C,D).

## Anti-biofouling polymers with special surface wettability for biomedical applications

As discussed in Section 2 above, various strategies to achieve different surface wettability can produce anti-biofouling ability for different biomedical applications with extensive alternative schemes. In this section, we will review the applications of anti-biofouling polymers with special surface wettability in typical biomedical fields both *in vivo* and *in vitro*, including cardiology, ophthalmology, and nephrology.

### Heart Valves in Cardiology

**FIGURE 7:**
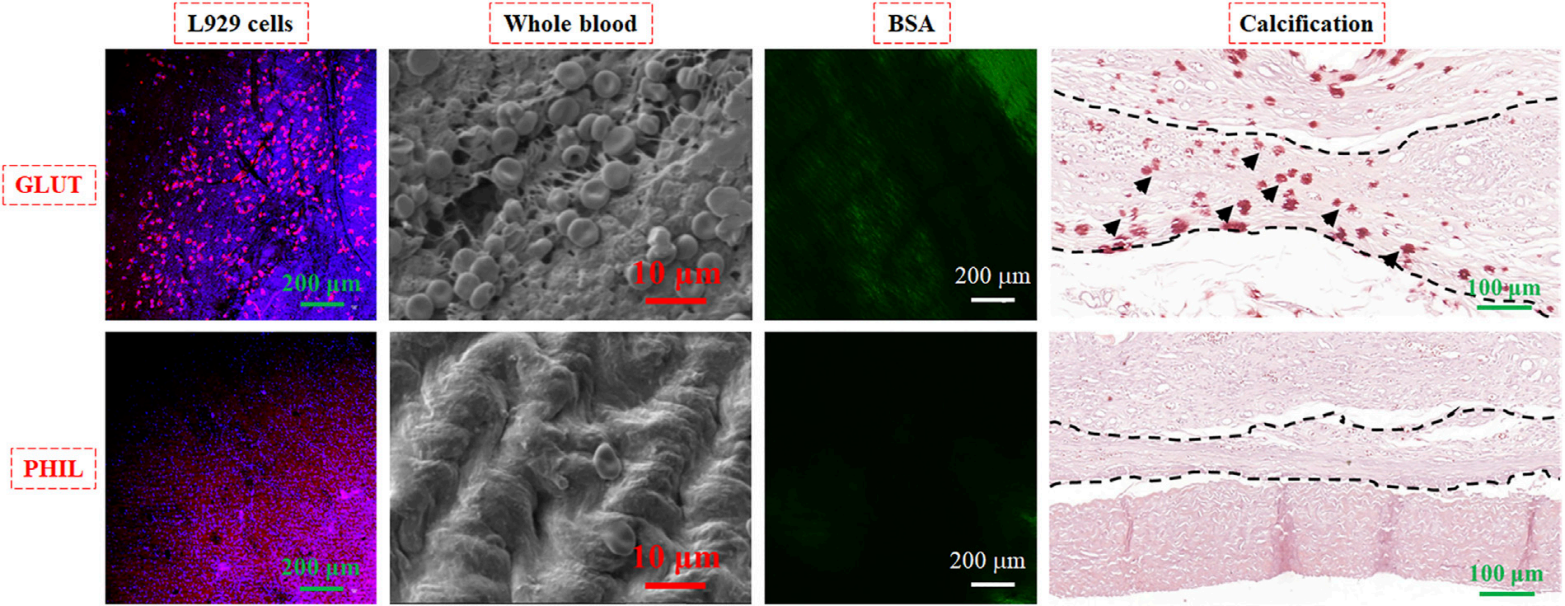
Anti-biofouling behavior of L929 cells, whole blood, FITC-labeled BSA, and calcification experiments on pristine BHV treated with GLUT and the sample treated with anti-biofouling coating (PHIL). Reprinted with permission from Ref. (Lei et al., 2021).

Although the bioprosthetic heart valve (BHV) has been used in clinical applications, there are still some complications, including calcification and thrombosis, which will shorten the service life of BHV. Hydrophilic polymers such as PAA are usually utilized to enhance the anti-biofouling actions of materials (Zhang et al., 2021c). Our previous article proposed a strategy to fabricate a hydrophilic-coated anti-biofouling BHV using PAA and PDMS in the inner and outer valves (Lei et al., 2021). We evaluated the anti-biofouling properties, including anti-coagulation, anti-cell adhesion, anti-calcification, and ability to resist BSA adsorption, both *in vivo* and *in vitro* (Figure 7). The anti-biofouling-coated sample (PHIL) was significantly better than the GLUT-treated control sample in various tests, including attachment of L929 cells, whole blood, FITC-BSA, and calcification. These results indicate the effectiveness of this method to produce hemocompatible biomedical materials with good anti-biofouling abilities.

**FIGURE 8:**
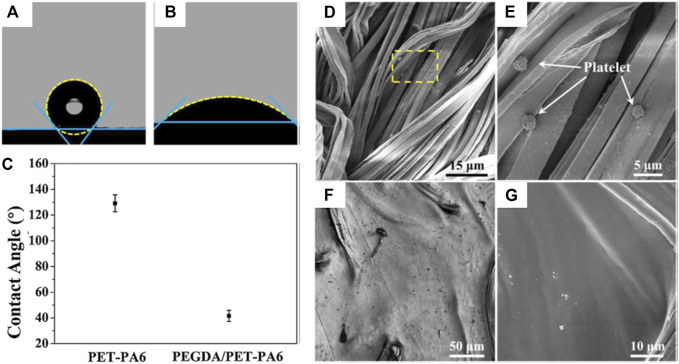
Profiles of water droplets on PET-PA6 fabric **(A)** and PEGDA/PET-PA6 composite **(B)**, and WCA results of the samples **(C)**. SEM micrographs showing platelet adherence on the PET-PA6 fabric **(D**,**E)** and PEGDA/PET-PA6 composites **(F**,**G)**. Reprinted with permission from Ref. (Guo et al., 2019). Copyright 2019, Elsevier B.V.

Polymeric heart valves have attracted much attention (Bezuidenhout et al., 2015; Guo et al., 2019; Kambe et al., 2019). Xing Zhang and coauthors reported that a composition of PEGDA hydrogels and polyethylene terephthalate/polyamide6 (PET-PA6) fabric (PEGDA/PET-PA6) was fabricated to form artificial heart valve leaflets (Guo et al., 2019). The WCA on the PET-PA6 fabric was about 129° (Figure 8A) but it decreased to about 41° (Figures 8B,C) after the introduction of PEGDA hydrogels, showing an obvious increase of surface hydrophilicity. After porcine platelet-rich plasma was cultivated for 2 h, a few platelets were seen on the PET-PA6 material (Figures 8D,E), while none were visible on the PEGDA/PET-PA6 composite (Figures 8F,G). Therefore, the increased hydrophilicity from the PEGDA hydrogels could enhance the anti-biofouling ability of the composite artificial heart valve leaflets with a low thrombogenic risk when interacting with blood.

### IOLs and CLs in Ophthalmology

**FIGURE 9:**
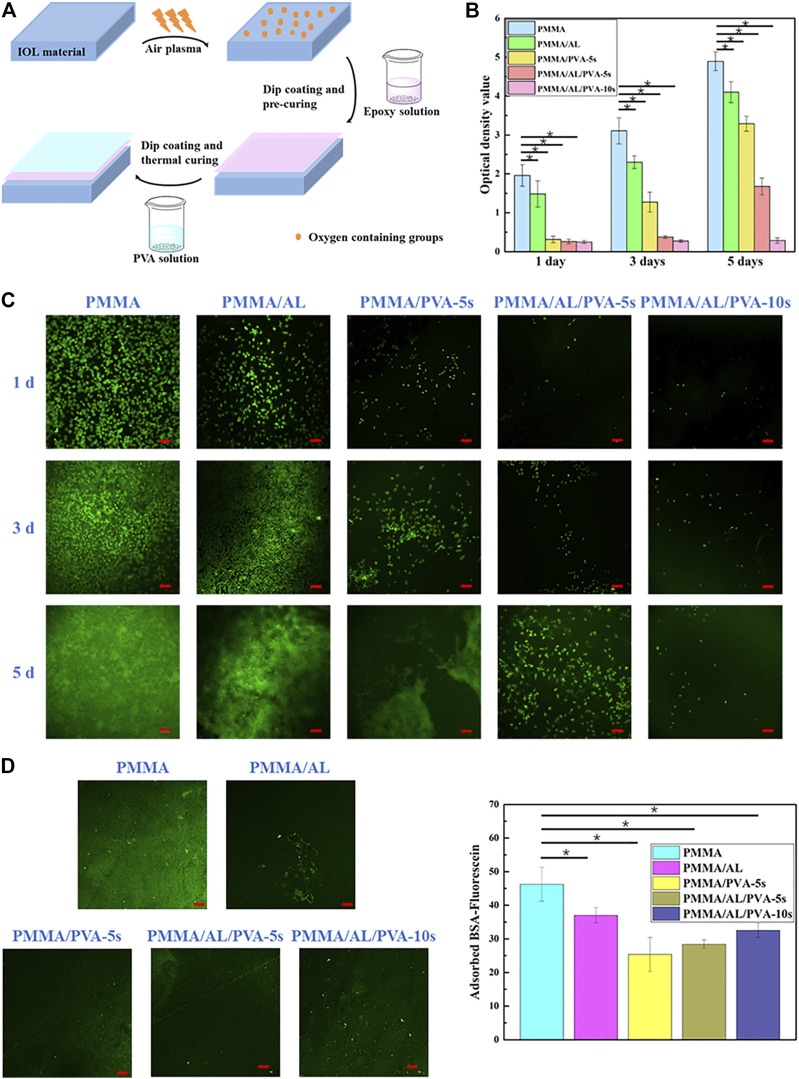
Fabrication process of hydrophilic PVA with an intermediate AL on PMMA IOL surface **(A)**. Cell proliferation measured by CCK-8 assay **(B)** and proliferation at 1, 3, and 5 days pre- and post-modification **(C)**. Fluorescent protein absorbance **(D, left)** and the fluorescence intensity **(D, right)** of FITC-BSA in different samples. Reprinted with permission from Ref. (Lan et al., 2021). Copyright 2021, Royal Society of Chemistry.

Biofoulant adhesion, including the attachment of bacteria, cells, or proteins, to devices such as IOLs can result in the failure of the implant. We fabricated a simple and economical PVA coating with or without the introduction of a “bridge.” The “bridge” comprised an intermediate adhesive layer (AL) to augment the interaction between the coating and the IOL material (Figure 9A) (Lan et al., 2021). Cell proliferation on the material was measured using CCK-8 assays (Figure 9B) and the adhesion of L929 cells measured by CLSM is shown in Figure 9C. The fluorescent protein adsorption performance and the fluorescence intensity of FITC-BSA on different samples were shown in Figure 9D. Increasing the PVA coating time to 10 s resulted in a reduction in the WCA to approximately 63°, in conjunction with augmented hydrophilicity and anti-biofouling action against both L929 cells and BSA. The coating prepared by AL “bridge” could greatly improve the mechanical stability of PVA coating on PMMA IOL surface to extend the lifetime of anti-biofouling ability, which could provide a new means of preparing a transparent hydrophilic anti-biofouling PVA coating applicable to IOLs.

**FIGURE 10:**
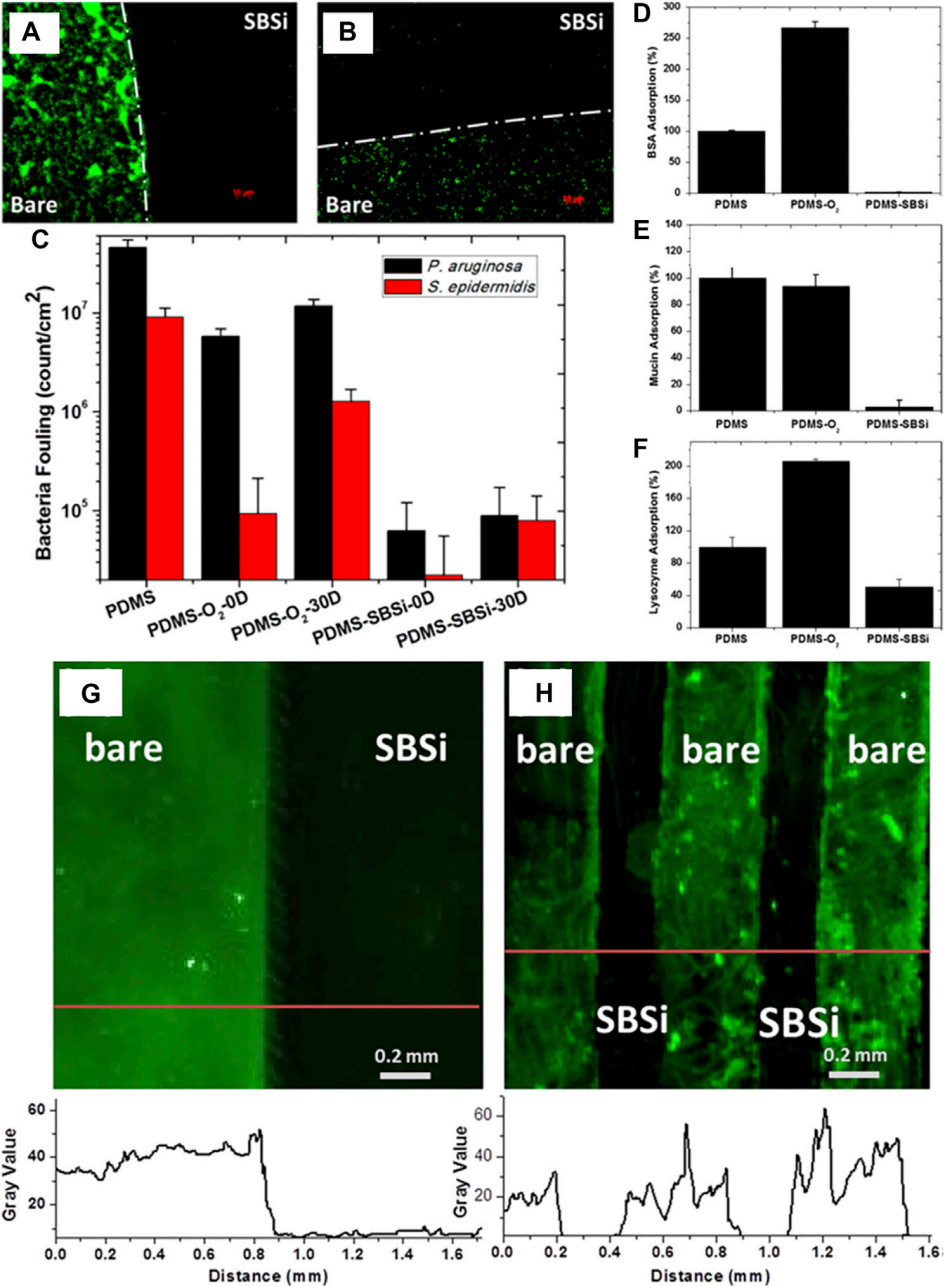
Fluorescence micrographs showing *P. aeruginosa* **(A)** and *S. epidermidis* **(B)** adsorption to partially modified PDMS. Quantification of adsorption on PDMS samples as a function of treatment and time **(C)**. Enzyme-linked immunosorbent assay (ELISA) measurements for adsorption of BSA **(D)**, mucin **(E)**, and lysozyme **(F)** on samples of PDMS, PDMS-O_2_, and PDMS-SBSi. Adsorption of SRB-encapsulated liposomes on SBSi-patterned PDMS samples prepared by elastomeric stencil **(G)** and microchannels **(H)**. Fluorescence intensities are indicated by red lines below the images. Reprinted with permission from Ref. (Yeh et al., 2014). Copyright 2014, American Chemical Society.

Besides the IOL, CLs are a common application in ophthalmology and the development of anti-biofouling CLs would ensure safety. Gongyan Liu and coauthors introduced the zwitterionic anti-biofouling carboxybetaine groups onto the surface of CLs to significantly increase their wettability and reduce their adsorption of bacteria and proteins (Liu et al., 2020c). Yiyan Yang and coauthors reported a series of polymers that were conjugated with adhesive catechol, anti-biofouling PEG, and hydrophobic urea/ethyl onto branched poly(ethylenimine). The CLs were coated by immersing in aqueous solutions of the modified polymers, and the coating was found to tolerate autoclaving, remaining on the device for its lifetime of approximately 7 days (Cheng et al., 2015). Silicone is widely utilized in biomedical devices, and the most commonly used silicone is PDMS as it is transparent, inert, inflammable, and non-toxic. Chun-Jen Huang and coauthors developed a stable superhydrophilic zwitterionic interface on PDMS by covalent silanization of sulfobetaine silane (SBSi) (Yeh et al., 2014). This was effective against biofouling by both *Pseudomonas aeruginosa* and *S. epidermidis* even after storage for 30 days at room temperature (Figures 10A–C), and the SBSi-modified commercially available silicone hydrogel CLs showed similar excellent anti-biofouling ability. Meanwhile, the adsorption of BSA, mucin, lysozyme (Figures 10D–F) and sulforhodamine B sodium (SRB)-encapsulated liposomes (Figures 10G,H) on SBSi-tailored PDMS showed an obvious decrease compared with that on pure PDMS.

### Urinary Catheters and Hemodialysis Membranes in Nephrology

Urinary catheters and hemodialysis membranes are the typical polymer materials used in nephrology. Various strategies to prevent bacterial adhesion and growth on medical devices have been developed. Poloxamers are nontoxic hydrophilic copolymers and Poloxamer 338 (P388) can be used to prevent the formation of biofilm and consequent infection. The anti-biofouling behavior was investigated by the adhesion of Ec5FSL and Ec9FSL *E. coli* on a segment of a hydrophilic P388-adsorbed silicone urinary catheter compared to an uncoated segment. Neither *E. coli* isolate was detected on the former due to the excellent anti-biofouling ability of hydrophilic P388 (Stirpe et al., 2020).

Hemodialysis membrane is another typical example of a nephrological application. PSF is frequently used for ultrafiltration membranes due to its stability under various conditions (Xie et al., 2015; Yin et al., 2015). A variety of surface modifications for ultrafiltration membranes have been investigated to combat biofouling. A novel zwitterionic molecule, MPDSAH, was grafted onto PSF membranes using benzophenone to increase their anti-biofouling actions (Yu et al., 2009). Measurement of WCA indicated the enhancement of membrane hydrophilicity by this modification. The passage of water was somewhat reduced by the modification, while adsorption of BSA was significantly reduced. The increase in anti-biofouling action was shown to be related to increased surface hydrophilicity (Yu et al., 2009). The immobilization of heparin on PSF allowed use of the membrane for dialysis. The higher the heparin density, the lower WCA and the platelet adherence. The flux of the heparin-modified membrane also recovered well after BSA filtration, indicating the improved anti-biofouling action of the heparin-modified membrane (Huang et al., 2011).

**FIGURE 11:**
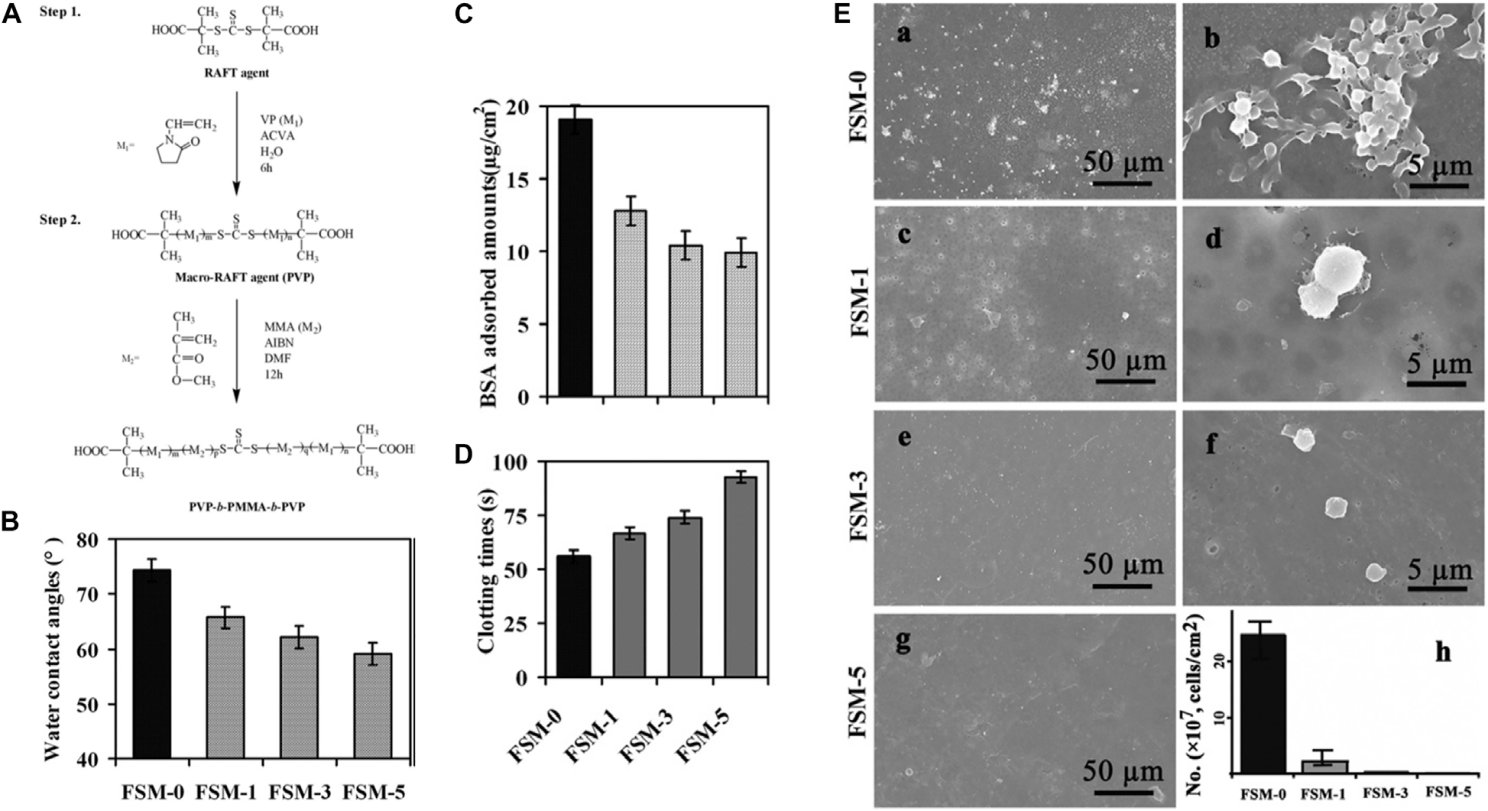
Synthesis of the PVP-*b*-PMMA-*b*-PVP block copolymer **(A)**. WCA of the modified membranes **(B)**. BSA adsorption **(C)**. Activated partial thromboplastin time **(D)**. SEM micrographs showing platelet adhesion (**E**, h, number of the adherent platelets on the membranes adsorbed from platelet-rich plasma estimated from the SEM pictures). Reprinted with permission from Ref. (Ran et al., 2011). Copyright 2011, Elsevier B.V.

PES is a typical PSF and is frequently used for hemodialysis membranes. Poly (citric acid)-grafted-MWCNT (PCA-g-MWCNT) was included as a nanofiller in PES to generate a hemodialysis mixed-matrix membrane (MMM) with improved hydrophilicity (from 77° to 56°) and anti-biofouling ability (Abidin et al., 2016). The passage of pure water and the resistance to BSA were increased as a result of the presence of numerous hydrophilic groups derived from PCA-*g*-MWCNT (Abidin et al., 2016). Changsheng Zhao and coauthors synthesized a hydrophilic triblock copolymer of PVP-*b*-PMMA-*b*-PVP *via* reversible addition-fragmentation chain transfer polymerization (Figure 11A) (Ran et al., 2011). After the introduction of the copolymer, the membranes showed a lower WCA (Figure 11B), lower BSA adsorption (Figure 11C), prolonged blood coagulation times (Figure 11D), and reduced platelet adhesion (Figure 11E). These results suggested that modifying the surface hydrophilicity of PES improves their anti-biofouling action, allowing the membranes to be used for blood purification, including hemodialysis (Ran et al., 2011).

**FIGURE 12:**
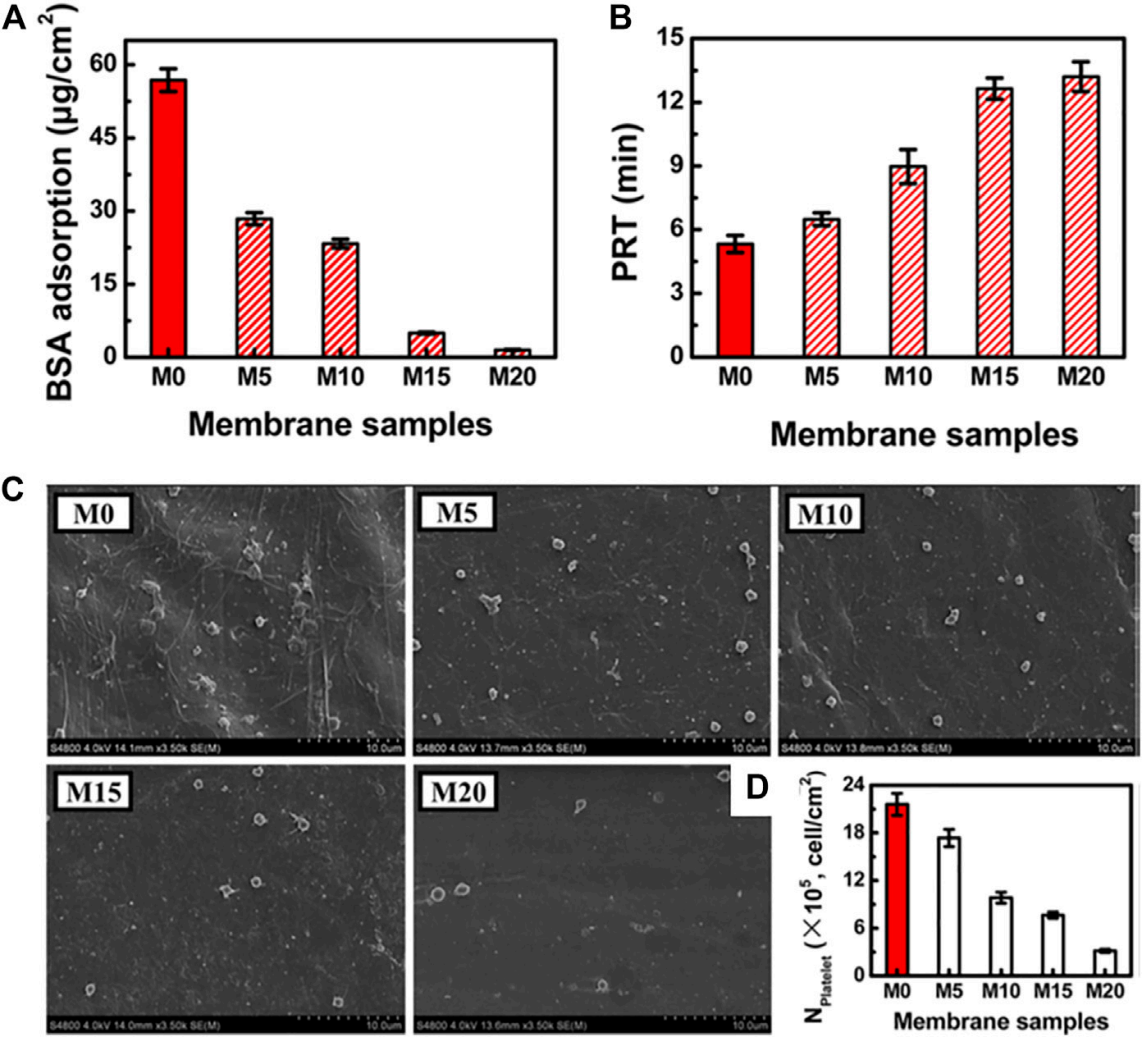
Hemocompatibility of M0 and PLA/PLA−PHEMA membranes (M5, M10, M15, and M20). Adsorption of BSA to membranes **(A)**. Plasma recalcification times for membranes **(B)**. SEM micrograph **(C)** and the number **(D)** of adherent platelets on the membrane surfaces. Reprinted with permission from Ref. (Zhu et al., 2015). Copyright 2015, American Chemical Society.

PHEMA, the simplest hydroxylated polymethacrylate, can impart an anti-biofouling character to surfaces. Lixin Xue and coauthors reported another hemodialysis membrane based on biobased and biodegradable PLA and PHEMA (Zhu et al., 2015). Anti-biofouling and hemocompatible PLA membranes were developed using different concentrations of PLA-PHEMA copolymers as the blending additive (M0 indicates pure PLA membrane, and M20 indicates 20 wt% copolymer). The results showed that PLA/PLA-PHEMA membranes with high PLA-PHEMA concentrations showed augmented hydrophilicity (WCA decreased from 75° for M0 to 60° for M20), water permeability, anti-biofouling (decreased BSA adsorption and platelet adhesion, Figures 2, 12A,C,D) and hemocompatibility (increased plasma recalcification time (PRT), Figure 12B). These findings indicate that PLA-PHEMA copolymers were effective in optimizing PLA membranes for hemodialysis applications (Zhu et al., 2015).

## Conclusion and Outlook

The ability of polymers to combat biofouling can be enhanced by the regulation of special surface wettability, including superhydrophilicity, hydrophilicity, hydrophobicity, and superhydrophobicity. In this review, we focus on the anti-biofouling polymers with superhydrophilicity, hydrophilicity and hydrophobicity but excluding superhydrophobicity which can be found in our previous review (He et al., 2021). Meanwhile, we just focus on the biomedical applications in cardiology, ophthalmology, and nephrology. Moreover, biofoulants mentioned in this review are focused on the usual bacteria, cells, and proteins. This review will provide a new perspective to promote the development of anti-biofouling polymers. Meanwhile, the anti-biofouling strategies reviewed in this manuscript will offer help for future researchers to choose suitable polymers for specific anti-biofouling applications. Considering the biomedical applications of anti-biofouling polymers, most research has focused on materials with superhydrophilicity or hydrophilicity which may be more achievable than hydrophobicity or superhydrophobicity. However, there is still some research that obtained excellent anti-biofouling polymers by hydrophobic manipulation. Therefore, exploring more anti-biofouling polymers with hydrophobic or superhydrophobic properties should be a research priority in the future to avoid the drawbacks of anti-biofouling polymers with hydrophilic properties. Meanwhile, there are some issues that should be paid more attention, that are discussed below.

### Exploring the Inherent Correlation Between the Anti-Biofouling and Surface Wettability

**FIGURE 13:**
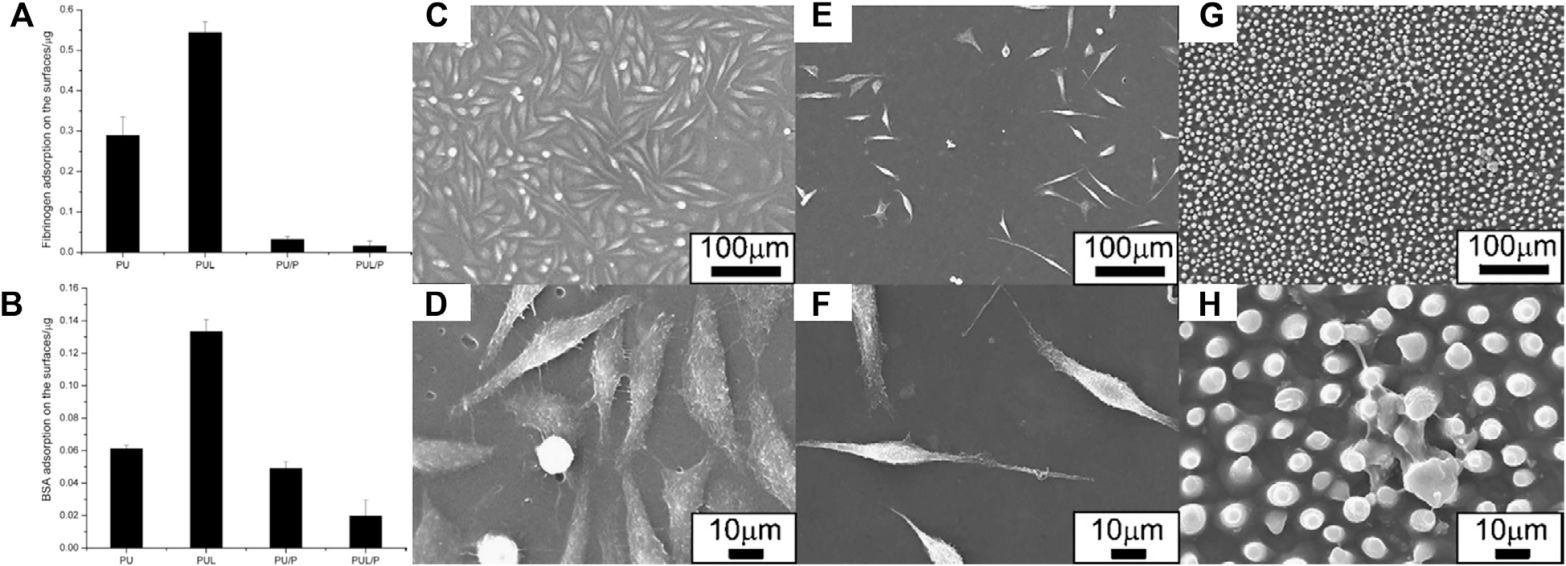
Adsorbed fibrinogen **(A)** and BSA **(B)** on PU, PU/P, PUL and PUL/P. SEM micrograph showing cell morphologies on PU **(C**,**D)**, PU/P **(E**,**F)**, and PUL/P **(G**,**H)**. Reprinted with permission from Ref. (Zheng et al., 2010). Copyright 2010, Elsevier B.V.

Protein adsorption depends not only on the hydrophilic or hydrophobic properties of materials, but also on topographical features, including surface curvature, roughness, and geometrical characteristics. There is research focusing on the effects of surface micro- or nano-typography on adsorption. In 1964, Curtis and Varde described the effects of surrounding topography on cells. It is generally accepted that both the topography and chemical characteristics of surfaces can influence the growth and properties of cells (Zheng et al., 2010). Hong Chen and coauthors fabricated lotus leaf-like polyurethane/Pluronic^®^ F-127 surface (PUL/P) by replica molding using a lotus leaf as the template (Zheng et al., 2010). When water droplets touched the superhydrophilic PUL/P surface, the drop spread rapidly with a WCA near zero, suggesting enhancement of the surface by the adoption of the lotus leaf-like structure compared to those without (PU/P). Adsorption of both BSA and fibrinogen was significantly lower on the PU/P surface (Figures 13A,B). Further reductions in adsorption were observed on the superhydrophilic PUL/P surface (Figures 13A,B). Experiments with L929 cells showed that cells adhered less to PU/P surfaces (Figures 13C–F). Meanwhile, cell adhesion to the superhydrophilic PUL/P was reduced with cells showing spherical shapes and diminished viability (Figures 13G,H). The superhydrophilic PUL/P thus appears to resist non-specific protein adsorption and cell attachment, with these effects deriving from both topographical and chemical structures. However, adsorption and adhesion to the hydrophobic lotus leaf-like polyurethane surface (PUL) were obviously enhanced compared with the hydrophilic PU, in apparent contradiction of the anti-biofouling strategies based on hydrophobic polymers discussed in section 2.3. Biofoulants adsorption and adhesion may be increased or decreased with the increased hydrophobicity. Therefore, the inherent correlation between the anti-biofouling and surface wettability was still non-uniform, and in-depth research should be applied. A comprehensive consideration and more quantitative research of the influence of chemical compositions and physical structures on the anti-biofouling ability should be under consideration.

### Design and Fabrication of Surfaces

**FIGURE 14:**
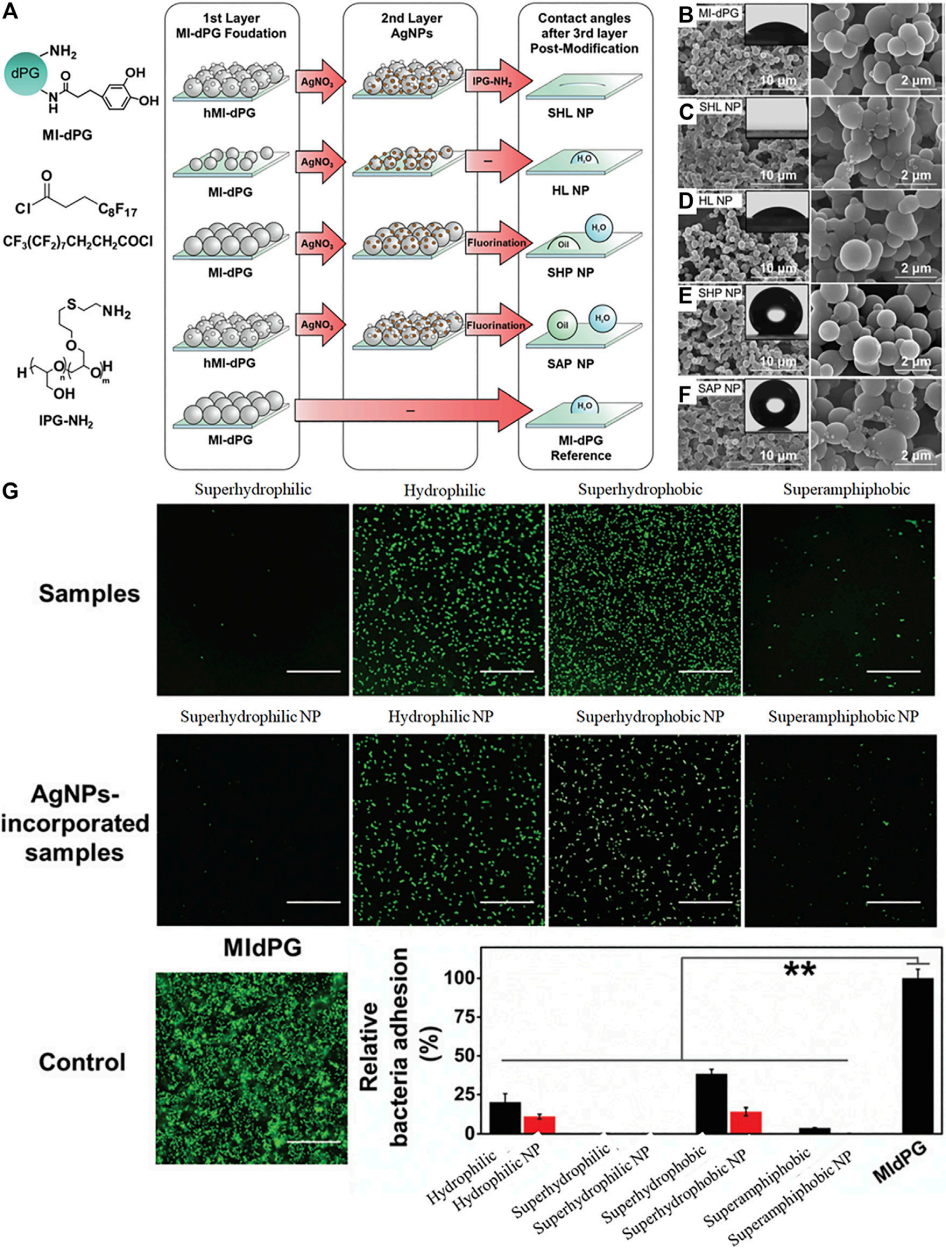
Fabrication process **(A)** of MI-dPG based coatings with different wettability characteristics and physical structures, MI-dPG **(B)**, superhydrophilic NP **(C)**, hydrophilic NP **(D)**, superhydrophobic NP **(E)**, superamphiphobic NP **(F)**. Quantification of bacterial attachment and their corresponding micrographs **(G)**. Reprinted with permission from Ref. (Li et al., 2019b). Copyright 2019, Royal Society of Chemistry.

Both chemical and physical properties influence the resistance of surfaces to biofoulants. This is borne out in natural antifouling materials (Magin et al., 2010). Rainer Haag and coauthors reported the effect of extreme wettability ranging from superhydrophilicity to superhydrophobicity on the antibacterial efficiency of an MI-dPG and silver nanoparticle (AgNPs) coating (Li et al., 2019b). As shown in Figure 14A, MI-dPG or hierarchical micro- and nanometer roughed MI-dPG (hMI-dPG) was formed by controlling surface polymerization and subsequent modification by AgNPs, linear polyglycerol (lPG-NH_2_) or fluorination. Different surface wetting properties containing superhydrophilic, hydrophilic, superhydrophobic, and even superamphiphobic wettability were achieved by different post-functionalization without obvious physical structural changes, demonstrated by WCA and SEM (Figures 14B–F). The resulting superhydrophilic polymer coatings were effective in repelling both *E. coli* and *S. aureus,* and the coating properties in relation to their antibacterial activities are shown in Figure 14G. The results indicated that the polymer coatings with superhydrophilic or superamphiphobic properties had good anti-biofouling ability but those with hydrophilic and superhydrophobic character showed less anti-biofouling ability.

Therefore, it is necessary to develop surfaces with quantifiable physical structures for optimization of anti-biofouling action. We previously reported a simple and mold-free technique for fabricating surfaces by 3D printing (He et al., 2017), which may assist in the precise design and manufacture of physical surfaces to combat biofouling (Mazinani et al., 2019).

### Anti-biofouling Polymers With Good Biocompatibility

Biocompatibility is defined as the ability of a material to perform with an appropriate host response in a specific application (Grainger, 1999; Barrère et al., 2008). It is an important issue for the chosen of anti-biofouling polymer types especially when these materials will be used in biomedical fields. If anti-biofouling polymers used *in vivo* are not biocompatible, they will elicit pernicious local or systemic inflammatory responses and induce the biomedical implants failed. Therefore, good biocompatibility should be paid more attention in the future researches of anti-biofouling polymers with special surface wettability.

### Long-Term Anti-Biofouling Polymers

Anti-biofouling surfaces are easily damaged by scratching, degradation, and rough handling. This damage destroys the surface characteristics leading to a loss of anti-biofouling activity. The development of robust and long-lasting anti-biofouling polymers remains a major challenge (Wu et al., 2019). Investigation of self-repairing materials, inspired by the repair mechanisms seen in natural organisms (Cai et al., 2014) would be advantageous to maintain and restore the properties of surfaces. This is a promising strategy for obtaining long-term and robust anti-biofouling surfaces (Wang et al., 2011; Chen et al., 2015a; Chen et al., 2015b; Chen et al., 2016; Liu and Guo, 2018; Wang et al., 2020).

## Footnotes

1
http://corrosion-doctors.org/Seawater/Fouling.htm

## Abbreviations

BSA, bovine serum albumin; CLs, contact lens; E. coli, *escherichia coli*; FITC-BSA, fluorescein isothiocyanate conjugate-bovine serum albumin; FITC-HSA, fluorescein isothiocyanate conjugate-human serum albumin; IgG, immunoglobulin G; IOL, intraocular lens; MI-dPG, mussel-inspired dendritic polyglycerol; MPDSAH, (3-(methacryloylamino)propyl)-dimethyl (3-sulfopropyl) ammonium hydroxide inner salt; PAA, poly(acrylic acid); PCBAA, poly(carboxybetaine acrylamide); PCBMA, poly(carboxybetaine methacrylate); PCL, polycaprolactide; PDMS, poly(dimethyl siloxane); PEG, poly(ethylene glycol); PEGDA, poly(ethylene glycol) diacrylate; PEO, poly(ethylene oxide); PES, poly(ether sulfone); PHEMA, poly(2-hydroxyethyl methacrylate); PLA, poly(lactic acid); PMMA, poly(methyl methacrylate); PPGL, homopolymer grafts from α-tert-butoxy-ω-vinylbenzyl-polyglycidol; PSBMA, poly(sulfobetaine methacrylate); PSF, polysulfone; PU, polyurethanes; PVA, poly(vinyl alcohol); PVP, poly(N-vinyl pyrrolidone); S. aureus, *staphylococcus aureus*; SEM, scanning electron microscopy; S. epidermidis, staphylococcus epidermidis; WCA, water contact angle.
