# Supplementary figures and images for: Anti-Biofouling Polymers with Special Surface Wettability for Biomedical Applications
Source: Front Bioeng Biotechnol. 2021 Dec 7;9:807357. doi: 10.3389/fbioe.2021.807357 (PMC8688920; doi:10.3389/fbioe.2021.807357)

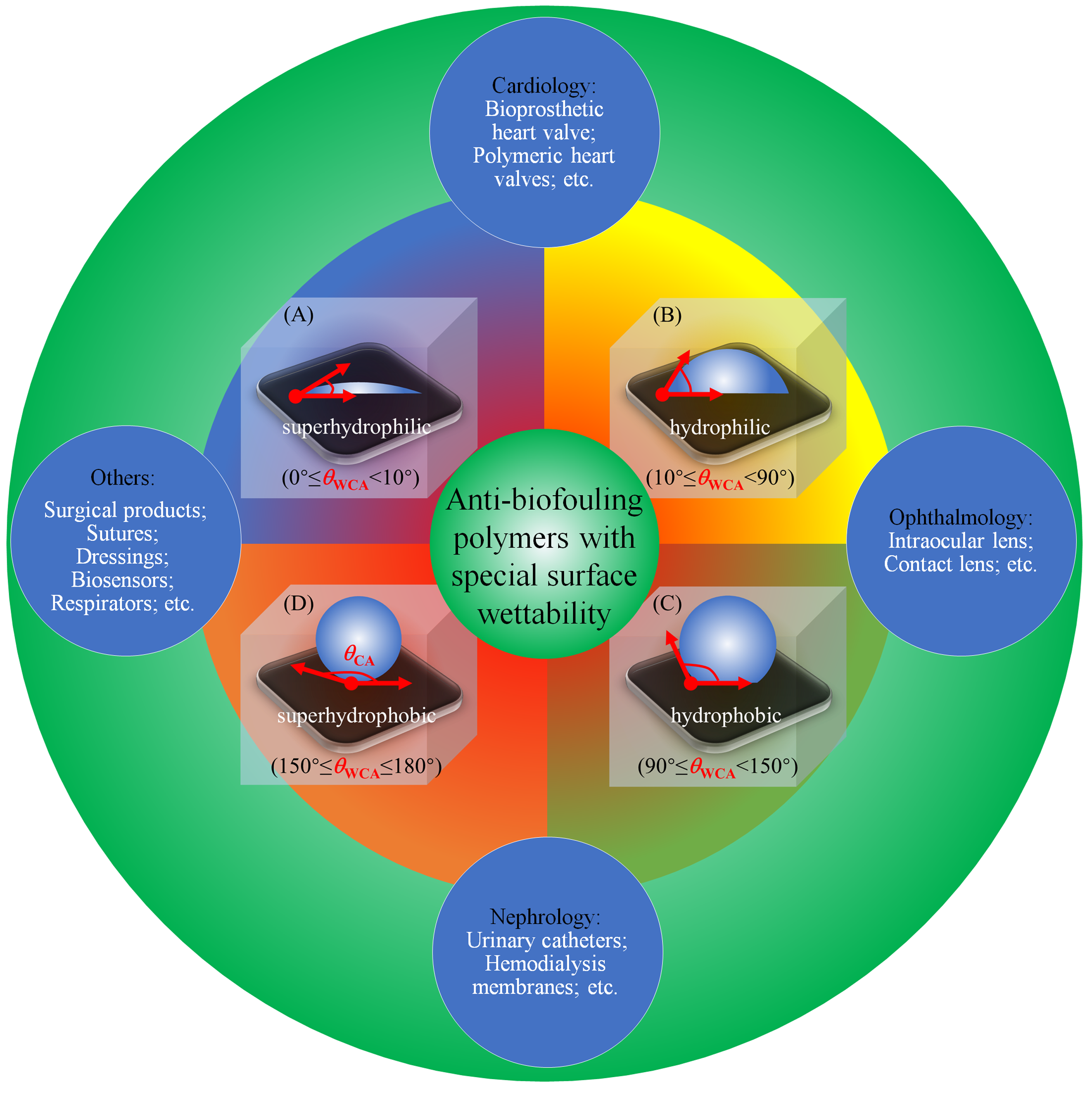

Supplement: Supplementary file 1 [file Image1.TIF]
